# Cortico-muscular coupling to control a hybrid brain-computer interface for upper limb motor rehabilitation: A pseudo-online study on stroke patients

**DOI:** 10.3389/fnhum.2022.1016862

**Published:** 2022-11-22

**Authors:** Valeria de Seta, Jlenia Toppi, Emma Colamarino, Rita Molle, Filippo Castellani, Febo Cincotti, Donatella Mattia, Floriana Pichiorri

**Affiliations:** ^1^Department of Computer, Control, and Management Engineering, Sapienza University of Rome, Rome, Italy; ^2^Neuroelectric Imaging and BCI Lab, IRCCS Fondazione Santa Lucia, Rome, Italy

**Keywords:** EEG, Electromyography (EMG), CMC, BCI—brain computer interface, hybrid BCI, stroke, neurorehabilitation, online classification

## Abstract

Brain-Computer Interface (BCI) systems for motor rehabilitation after stroke have proven their efficacy to enhance upper limb motor recovery by reinforcing motor related brain activity. Hybrid BCIs (h-BCIs) exploit both central and peripheral activation and are frequently used in assistive BCIs to improve classification performances. However, in a rehabilitative context, brain and muscular features should be extracted to promote a favorable motor outcome, reinforcing not only the volitional control in the central motor system, but also the effective projection of motor commands to target muscles, i.e., central-to-peripheral communication. For this reason, we considered cortico-muscular coupling (CMC) as a feature for a h-BCI devoted to post-stroke upper limb motor rehabilitation. In this study, we performed a pseudo-online analysis on 13 healthy participants (CTRL) and 12 stroke patients (EXP) during executed (CTRL, EXP unaffected arm) and attempted (EXP affected arm) hand grasping and extension to optimize the translation of CMC computation and CMC-based movement detection from offline to online. Results showed that updating the CMC computation every 125 ms (shift of the sliding window) and accumulating two predictions before a final classification decision were the best trade-off between accuracy and speed in movement classification, independently from the movement type. The pseudo-online analysis on stroke participants revealed that both attempted and executed grasping/extension can be classified through a CMC-based movement detection with high performances in terms of classification speed (mean delay between movement detection and EMG onset around 580 ms) and accuracy (hit rate around 85%). The results obtained by means of this analysis will ground the design of a novel non-invasive h-BCI in which the control feature is derived from a combined EEG and EMG connectivity pattern estimated during upper limb movement attempts.

## Introduction

Brain-Computer Interface (BCI) systems for motor rehabilitation can improve functional outcome in stroke patients directly decoding the brain activity of the users (e.g., via electroencephalography, EEG) and providing them an online feedback on performance (Silvoni et al., 2011; Ramos-Murguialday et al., 2013; Pichiorri et al., 2015; Chaudhary et al., 2016; Mrachacz-Kersting et al., 2016; Biasiucci et al., 2018; Raffin and Hummel, 2018). Most BCIs target upper limb motor rehabilitation (Pichiorri et al., 2015), being the primary therapeutic goal in stroke rehabilitation to maximize patients’ functional recovery and reduce long-term disability (Coscia et al., 2019). Such rehabilitation approach focuses exclusively on brain activity and induces brain plasticity guiding the functional motor system reorganization after stroke (Daly and Wolpaw, 2008; Pichiorri and Mattia, 2020).

Along the process of motor recovery after stroke, several abnormalities in upper limb function have been described such as muscle weakness and spasticity, abnormal muscle co-activation, increased activity of the antagonist muscles (Levin et al., 2000; Miller and Dewald, 2012; Silva et al., 2014; Chen et al., 2018). Electromyography (EMG) can be used to monitor the residual or recovered muscular activity along the rehabilitation processes (Hesam-Shariati et al., 2017; Steele et al., 2020) and EMG-related features can be exploited to avoid the reinforcement of such maladaptive changes. Hybrid BCIs (h-BCIs) include peripheral signals such as EMG, in addition to brain signals, as control features (Choi et al., 2017) and they have mostly been developed to improve the classification performance of the system as in assistive BCIs (Leeb et al., 2011; Müller-Putz et al., 2015; Riccio et al., 2015; López-Larraz et al., 2018). Such devices usually combine the EEG and EMG feature in the classification stage, meaning that each feature (brain and muscular) is calculated separately and combined sequentially or simultaneously using a balanced weight or Bayesian fusion approach to better control the assistive device (Leeb et al., 2011; Lalitharatne et al., 2013). Only recently, h-BCIs (EEG-EMG) have been studied specifically for post-stroke upper limb rehabilitation (Chowdhury et al., 2019; Guo et al., 2022).

In this framework, Cortico-Muscular Coupling (CMC), which measures the synchronization between central and peripheral activation (Mima and Hallett, 1999), could be used as a hybrid feature to detect in real-time movement attempts and to train the physiological brain control over muscles activity (volition). Such a feature merges information from EEG and EMG signals depicting the functional communication between brain and muscles. Several studies conducted in stroke have shown CMC alterations in both the acute and chronic phases (Mima et al., 2001; von Carlowitz-Ghori et al., 2014; Guo et al., 2020), mainly represented by a decrease in CMC peak which has been correlated with functional recovery (Krauth et al., 2019). In a recent study on healthy subjects, we provided evidence that CMC features extracted from multiple EEG-EMG pairs can discriminate offline different simple hand movements, such as finger extension and grasping, from rest condition (Colamarino et al., 2021). CMC values have been already used as inputs of a h-BCI to discriminate online right-vs.-left hand grasp movement in both healthy subjects and hemiparetic stroke patients (Chowdhury et al., 2019). To our knowledge, these studies had neither assessed the ability of CMC to detect movement attempts from rest condition nor optimized the online CMC-based movement classification pipeline finding the parameters that allow the best trade-off between accuracy and speed.

Here, we evaluated the feasibility of real-time extraction of CMC features suitable for movements vs. rest classification, and thus to control a h-BCI system. We analyzed the data of 13 healthy (CTRL) and 12 stroke (EXP) participants during executed (CTRL and EXP unaffected arm) and attempted (EXP affected arm) simple hand movements simulating a real-time approach (i.e., pseudo-online) to optimize the choice of the parameters in the real-time CMC algorithm that allow the best trade-off between classification performances and classification speed. Indeed, together with a high classification accuracy, also a short time for the BCI to detect a movement should be pursued, in order to lead to significant plasticity induction and functionally relevant improvement in agreement with Hebbian associative learning theory (Hebb, 1949; Mrachacz-Kersting et al., 2016). For this reason, different updating factors of the CMC computation (shifts) during the trial, as well as different number of consecutive movement predictions to accumulate for a final classification decision, were tested in terms of performance and time for detection. Once identified the best parameters to be used in the real-time CMC approach, we compared classification accuracy and speed obtained in stroke participants between different movements accomplished with affected and unaffected hands, separately.

## Materials and methods

### Participants

Thirteen right-handed healthy subjects (9 females/4 males, age 48.5 ± 19.3 yo) and 12 patients (7 females/5 males, age 53.8 ± 18 yo, months from event 5.3 ± 3.5, lesion side: 7 left/5 right) with clinically diagnosed stroke participated in the study. Healthy subjects did not present any evidence or known history of neuromuscular disorders, whereas for stroke patients the following inclusion criteria were applied: (1) a history of first-ever unilateral, cortical, subcortical, or mixed stroke, caused by ischemia or hemorrhage (confirmed by magnetic resonance imaging), that occurred at least 6 weeks prior to study inclusion; (2) upper limb hemiplegia/hemiparesis that was caused by the stroke; and (3) age between 18 and 80 years. The exclusion criteria were the presence of chronic disabling diseases, such as orthopedic injuries that could impair reaching or grasping; spasticity of each segment of the upper limb scored higher than 4 on the Modified Ashworth Scale (MAS) (Bohannon and Smith, 1987).

Clinical and functional evaluation was performed by expert physiotherapists before data acquisition (same day). The upper extremity section of the Fugl-Meyer Assessment scale (FMA-UE, motor domains only ranging from 0—maximum impairment to 66—normal function) (Fugl-Meyer et al., 1975) was performed to describe the residual arm function of stroke participants. The National Institute of Health Stroke Scale (NIHSS) was performed to assess general impairment derived from stroke (Goldstein et al., 1989). Handedness was assessed in all participants by means of the short form of the Edinburgh Handedness Inventory (EHI) (Oldfield, 1971). Details about patients’ demographical and clinical data are reported in Supplementary Table 1. The study was approved by the local ethics board at Fondazione Santa Lucia, IRCCS, Rome, Italy (CE PROG.752/2019), the protocol was written according to the Helsinki Declaration and all the participants signed an informed consent.

### Experimental design

**FIGURE 1:**
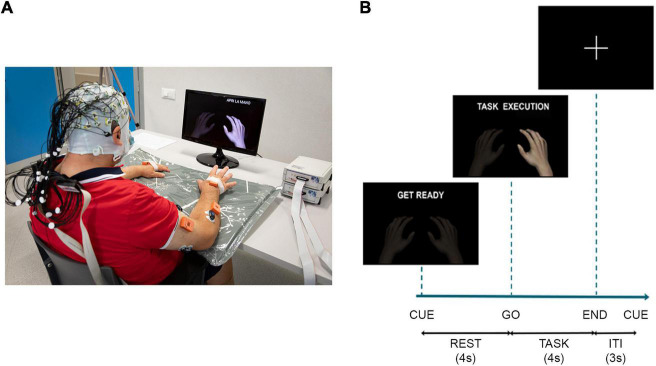
**(A)** Schematic of the experimental setup: participants wore an EEG cap over the scalp and EMG sensors over the upper limbs, they watched a screen placed 1 m in front of them on which a cue provided information on when to perform/attempt the movement. In addition to EEG and EMG data, the recording included also kinematic data. They were collected at 100 Hz by means of 8 IMUs (MTw Awinda, Xsens Technologies, The Netherlands). The IMUs were placed by a double-sided medical tape on the following anatomical points: hand, mid forearm, mid arm of both upper limbs, over the clavicular notch and at the lumbar vertebrae level. The participant signed a written informed consent to the collection and use of his images. **(B)** Timeline of the experiment for task trials with the instructions provided to the participants on the screen, ITI, inter-trial-interval.

We simultaneously recorded EEG and EMG data sampled, respectively, at 1,000 and 2,000 Hz. Sixty-one active electrodes arranged according to an extension of 10-20 system (reference on left mastoid and ground on right mastoid) were used to acquire the EEG data from the scalp by means of BrainAmp amplifiers (Brain Products GmbH, Germany^1^), impedances were kept below 5 kΩ. Surface EMG data were recorded through Pico EMG sensors (Cometa S.r.l., Italy^2^) from 16 muscles collected in bipolar fashion: extensor digitorum (ED), flexor digitorum superficialis (FD), lateral head of the triceps muscle (TRI), long head of the biceps brachii muscle (BIC), pectoralis major (PEC), lateral deltoid (Lat_DELT), anterior deltoid (Ant_DELT) and upper trapezius (TRAP) of both sides (L: left, R: right). EMG sensors were placed according to the guidelines reported in Barbero et al. (2012). A TriggerBox (Brain Products GmbH, Germany) was adopted to synchronize the EEG and EMG acquisition. The quality of EEG and EMG signals was visually checked prior to beginning the recordings and continuously monitored afterward. The experiment consisted of 4 runs, with a break among them, in which the participants were asked to perform/attempt finger extension (Ext) and grasping (Grasp) with the right and the left hand separately (unaffected-UH and affected-AH hand for stroke participants). The Maximum Voluntary Contraction (MVC) was recorded for each muscle at the beginning of the experiment for 5 s and the MVC values of the target muscles (ED and FD of both sides) were computed right after. During the experiment, all participants were seated in a comfortable chair with adjustable seat height and with their forearms placed on the table. Visual cues were presented on a screen on the desk in front of them via Matlab’s Psychtoolbox.^3^ The paradigm was administered using a block-design structure where the four runs were randomly ordered across participants. Each run comprised 40 trials equally divided in task (8 s duration) and rest (4 s duration) condition, presented to the participants according to a pseudo-random sequence which did not allow more than two consecutive task or rest trials and two consecutive rest trials at the beginning of the run to avoid fatigue and lapse in attention, respectively. The inter-trial-interval, during which a fixation cross was displayed in the middle of the screen, was set to 3 s. During rest trials participants had to stay relaxed for 4 s, whereas task trials began with 4 s of preparatory period, after which a go stimulus occurred, and the participant had to perform the task for 4 s (Figure 1). Participants were instructed to perform the movement as fast as they could and hold it at approximately 15% of the MVC of the target muscle until the end of the trial. Before starting the experiment, participants were asked to perform some repetitions guided by the experimenter, to understand how to perform the task at the desired activation level of 15% with respect to MVC of the target muscle. Subjects’ EMG level of activation of the target muscle normalized by its MVC was monitored by the experimenter via the EMG acquisition software (EMG and Motion Tools, Cometa S.r.l., Italy) during the entire experiment and indications on the muscular performance were given to the participants at each trial, when different from those requested. Stroke participants attempted the movement with their paretic hand to the best of their own residual ability.

### Pre-processing

EEG data were band-pass filtered 3–60 Hz whereas EMG signals were downsampled to 1,000 Hz and band-pass filtered 3–500 Hz. A notch filter at 50 Hz was applied to remove power-line artifacts on both signals, task trials were segmented in 8 s epochs from the cue onset, while rest trials were segmented in 4 s epochs from the cue onset. A subset of EEG channels over the sensorimotor area (FC5, FC3, FC1, FCz, FC2, FC4, FC6, C5, C3, C1, Cz, C2, C4, C6, CP5, CP3, CP1, CPz, CP2, CP4, CP6, P5, P3, P1, Pz, P2, P4, P6) was considered for the purposes of this study. Indeed, with the ultimate aim of successful online control, a low number of electrodes is desirable to improve the usability of the system, while the localization of the EEG electrodes over the sensorimotor areas ensures the use of physiologic features for movement detection. The epochs extracted from the trials and related to the subset of channels were then checked for compliance to the instruction and presence of artifacts in the EEG and EMG signals. We identified as non-compliant all the trials labeled as “Rest” in which participants moved or trials labeled as “Task” where subjects missed the instruction and did not perform the task. Such non-compliant trials were removed from the analysis. Regarding the artifacts management, we adopted two different criteria for the identification of artifacts in EEG and EMG signals. The EEG signals exceeding in absolute value the threshold of 100 μV were considered as artifactual. If artifacts were detected in more than one channel the trial was rejected; otherwise a spherical interpolation was performed to replace the noisy channel with a weighted average of its neighbors. A semi-automatic approach was used to detect the artifacts in the EMG signals: a statistical criterion based on the comparison between the EMG characteristics (Roland, 2020) of each trial and the median EMG characteristics of all trials (reference characteristic) was applied separately for task and rest condition. Once the EMG artifacts were detected by the statistical criterion, trials were visually inspected and validated for rejection.

EEG channels were interpolated due to presence of artifacts on average in 1% of trials for the movements performed by healthy participants. No channels were interpolated for the movements performed with the unaffected hand by stroke participants, whereas one EEG channel was interpolated on average in 1% of trials for the movement attempted with the affected hand. One stroke participant was excluded from Ext movements analysis due to the rejection of more than 50% of the trials (*n* = 11 for Ext movements analysis in stroke participants). After rejection of non-compliant and artefactual trials, the number of trials for healthy participants was on average 18.63 and 18.58 in task and rest condition, respectively. For stroke participants, on average 17.74 task trials and 17.91 rest trials were considered for the following analyses.

Pre-processing of EEG data was computed by means of Vision Analyzer 1.05 software (Brain Products GmbH, Gilching, Germany) while all the other steps described above were performed using custom codes developed in Matlab R2019a (The MathWorks, Inc., Natick, Massachusetts, USA).

### EMG onset detection

The EMG data of the target muscle (ED for Ext movements and FD for Grasp movements) have been processed to obtain the EMG onset for each task trial. The continuous raw EMG data were band-pass filtered in the range 30–300 Hz and a Teager–Kaiser energy operator was applied to improve Signal to Noise Ratio (SNR) and minimize erroneous EMG onset detection (Solnik et al., 2010). Signals were rectified and low pass filtered at 50 Hz. Then, EMG data were segmented in the 8 s-task trials and the EMG onset was identified applying the Hodges e Bui algorithm (Hodges and Bui, 1996) on EMG envelope for each task trial. Results were validated by visual inspection.

### CMC offline analysis

After the EEG/EMG preprocessing, we conducted an offline analysis with the following aims to: (i) identify the characteristic frequency of EEG-EMG coupling in beta band (13–30 Hz) for each EEG-EMG pair; (ii) select the most powerful CMC features in discriminating each movement from rest and (iii) assess offline the performances of CMC-based approach in movements detection against rest.

The data used for each participant in the offline analysis referred to a time interval of 1 s-length equal to the window [5–6]s in the task trials and [2–3]s in the rest trials. For those two intervals, the CMC between EEG signal and the rectified EMG signal (de Seta et al., 2021) was computed in the range 0–60 Hz as in Colamarino et al. (2021).

#### CMC characteristic frequency extraction

In this work, we considered as frequency band of interest the beta band (13–30 Hz), since previous studies identified it as the typical band for CMC (Liu et al., 2019). The CMC across trials was computed using 1 s-Hann windows with no overlap and the characteristic frequency for each EEG-EMG pair was extracted, as the frequency showing the highest CMC value in the beta band range during task trials (Colamarino et al., 2021). The computation was repeated for each movement and each participant. The single-trial CMC values obtained for each EEG-EMG pair at the characteristic frequency were considered for further analyses as feature space. For the single-trial CMC computation we used Welch periodogram with segments of 250 ms, 50% of overlap and tapered by means of the Hann window.

#### Feature selection

Since the number of features used for the classification impacts on the computational cost and the number of physical electrodes required to collect the data, the feature selection approach was used to choose two EEG-EMG pairs to be considered in the analysis. The original feature space (described in section “Cortico-muscular coupling characteristic frequency extraction”) was reduced by considering only the EEG-EMG pairs characterized by the EMG channel over the target muscle (ED for Ext movements, FD for Grasp movements) and by the EEG channels placed over the sensorimotor strip of the hemisphere contralateral to the hand involved in the task (ipsilesional hemisphere for the movement attempted with the paretic hand of stroke participants). Feature selection was performed by ranking the remained CMC features according to their discriminant power by Fisher criterion (Lal et al., 2004) and selecting the two most discriminant ones. This allowed to reduce the computational cost and achieve real-time movement detection.

For each movement and participant, the feature space was reduced to a 2-dimensional feature space, consisting of 40 observations (20 trials × 2 conditions, i.e., task and rest).

#### Binary classifier training

A 10-iteration cross-validation approach was used for offline detection of each movement vs. rest in both healthy and stroke participants. In each iteration, we used 80% of the observations (half labeled as task and half as rest) as training set, whereas the remaining 20% were used as testing set. A Support Vector Machine (SVM) classifier with a linear kernel was used as classification model on the reduced features space (see section “Features selection”). If the difference between the number of task and rest trials (after rejection of artefactual or non-compliant trials) was equal or higher than three, the two classes were balanced randomly selecting the same number of observations. The offline performances were assessed using the following classification metrics: Area Under the receiver operating characteristic Curve (AUC), accuracy, sensitivity and specificity (Chu, 1999; Fawcett, 2006).

### CMC pseudo-online analysis

The pseudo-online analysis was conducted using a sliding window approach mimicking the data reading from the temporary buffer of the amplifier used in the online acquisition of biological signals. We considered sliding windows of 1 s duration updated along the trial of a certain number of samples (shift parameter) to be varied in the study. The selected shifts were: 125, 250, and 500 ms. For each participant, movement and window in a trial, we computed single-trial CMC values in beta band for the two EEG-EMG pairs selected in the offline analysis. The CMC trend along the trial duration was then analyzed for the different shift values in the healthy participants with the aim to identify the best parameters to be used in the future online analyses. The pseudo-online analysis for stroke participants was conducted only for the best shift value identified in the analysis on healthy participants.

#### Identification of best shift value in data from healthy participants

In order to identify the best shift value to be used in the sliding window approach for CMC computation, we extracted movement onset from CMC trends along trial (in brief CMC onset) and compared it with the one extracted from EMG signal (in brief EMG onset), considered as the temporal reference for the beginning of the movement execution. The CMC onset was identified with a double-threshold criterion: we extracted the statistical threshold (95th percentile) from the distribution built considering all the CMC values of rest trials and the CMC onset was identified as the time point in which CMC values during task trials were above the statistical threshold in a temporal window equal or longer than 500 ms. The CMC onset was thus computed for each participant, movement type, shift and trial considering the CMC values of the EEG-EMG pair with the best CMC feature according to Fisher’s criterion.

For each trial, on the basis of the comparison between the EMG onset and the CMC onset we identified the following cases:

– True Detection (TD) if CMC onset was delayed with respect to the EMG onset
– False Detection (FD) if CMC onset was anticipated with respect to the EMG onset
– No Detection (ND) if no CMC onset was detected in presence of an EMG onset

The occurrence of TD, ND and FD across trials normalized for the total number of trials was computed for each participant, movement type and shift. These three performance parameters were flanked by a fourth one, the Mean Delay (MD) obtained as the temporal difference (in seconds) between the CMC onset and EMG onset only in TD case.

To identify the best value for shift parameter maximizing both accuracy and speed in CMC-based movement detection in the four movements analyzed, we computed two 2-way repeated measures ANOVA (rmANOVA) considering as within main factors the MOVEMENT (4 levels: ExtL, ExtR, GraspL, GraspR) and the SHIFT (3 levels: 125, 250, 500 ms) and as dependent variables the TD and the MD parameters, separately. ND and FD were not included in the statistical analysis due to their low rates obtained in almost all the participants. The statistical significance level for all tests was set to 0.05 and the Duncan’s *post-hoc* test was performed to assess differences among the levels of the within factors. A shift value of 125 ms resulted to achieve the highest performances (highest TD and lowest MD—see Results section “Identification of best shift value in data from healthy participants”) and was therefore used for further analyses.

#### Movement classification in healthy participants

To test the ability of CMC features in discriminating movements from rest condition in real-time, a single-subject pseudo-online validation was firstly performed in healthy participants. The same feature space used in the offline approach (see section “Features selection”) was adopted for the pseudo-online analysis. An adaptation of the Leave-One-Out Cross Validation was used to test the classification model for task trials in the pseudo-online approach. For each movement and participant, *N* different SVM classifiers (where *N* is the number of task trials) were trained excluding one task trial at a time from the training phase (training set observations = *N*_*tot trial*_ − 1) and tested on the excluded trial divided in 57 consecutive windows of 1 s with 125 ms of overlap (total number of observations in testing phase equal to 57 for each leave-one-out iteration).

The pseudo-online classification performances were evaluated considering as:

– True Positive (TP) when at least M consecutive sliding windows after the EMG onset of a task trial were predicted as task condition.
– False Positive (FP) when at least M consecutive sliding windows before the EMG onset of a task trial were predicted as task condition.
– False Negative (FN) when no M consecutive windows were predicted as task condition.

Here, the M parameter is the accumulation factor for which we tested three different values (1 – no accumulation, 2 and 3 windows) to identify the best trade-off between classification accuracy and speed. The following metrics were computed according to the number M of windows to be accumulated before a final movement detection:


(1) $$H\,i\,t\,r\,a\,t\,e=\frac{T\,P}{N}$$

(2) $$F\,a\,l\,s\,e\,P\,o\,s\,i\,t\,i\,v\,e\,R\,a\,t\,e\,\left(F\,P\,R\right)=\frac{F\,P}{N}$$

(3) $$F\,a\,l\,s\,e\,N\,e\,g\,a\,t\,i\,v\,e\,R\,a\,t\,e\,\left(F\,N\,R\right)=\frac{F\,N}{N}$$

(4) $$M\,e\,a\,n\,D\,e\,l\,a\,y\,\left(M\,D\right)=\frac{T_{M}-E\,M\,G_{o\,n\,s\,e\,t}}{T\,P}$$

where *N* is the number of task trials and *T*_*M*_ is the time after which *M* consecutive task predictions have been accumulated.

To evaluate the differences in the above metrics among the number M of consecutive windows, four 1-way rmANOVA were performed using as within main factor M (3 levels: *M* = 1, *M* = 2, *M* = 3) and as dependent variable the hit rate, the FPR, the FNR and the mean delay, separately. The Duncan’s *post-hoc* analysis was held to assess differences among the different levels of the within factor and the significant level was set to 0.05.

#### Movement classification in stroke participants

To evaluate whether the results obtained in healthy participants could be confirmed for movements performed/attempted by stroke patients, the same pseudo-online analysis described in section “Movement classification in healthy participants” was performed on data from stroke participants for each movement type. In particular, the CMC computation was performed with a sliding window approach considering windows of 1 s duration and a shift of 125 ms. Classification performances expressed in terms of hit rate, FPR and MD were firstly evaluated in the stroke participants group and then compared between movements performed with the affected and the unaffected hand by a paired *t*-test, considering only the 11 stroke participants analyzed during both Ext and Grasp movements. The significance level for all tests was set to 0.05. FNR was evaluated but not included in the comparison due to the low values obtained in all participants and movement types.

## Results

### CMC offline analysis

Offline classification performances of the movement vs. rest classifier based on two CMC features are shown in Supplementary Tables 2, 3 for healthy and stroke participants, respectively. Average AUC across healthy participants were ranging from 0.98 to 1.00, whereas slightly lower performances were achieved in stroke participants with AUC ranging from 0.93 to 0.98.

### CMC pseudo-online analysis

#### Identification of best shift value in data from healthy participants

**FIGURE 2:**
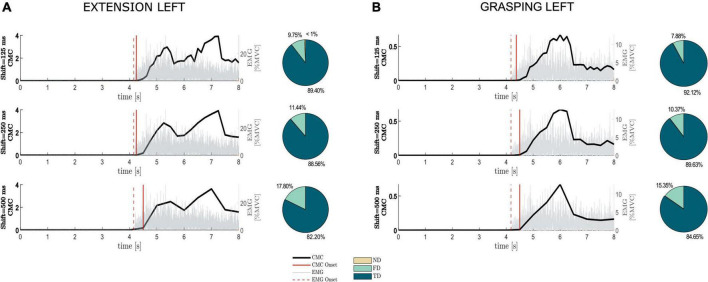
Impact of the shift value used in the sliding-window approach on the detection of the movement onset based on CMC (CMC onset). The average CMC and EMG trends across trials, considering the first EEG-EMG pair identified by Fisher criterion and the target muscle, respectively, were reported along trial duration for different shift values separately for extension and grasping of the left hand, (**A,B**, right panel), respectively, in one representative healthy participant (similar results were obtained for right-hand movements). Dashed vertical line represents movement onset detected from EMG (EMG onset), whereas continuous vertical line stays for detected CMC onset. Each graph is flanked by a pie chart reporting the percentages of No Detection (ND), False Detection (FD) and True Detection (TD) obtained on average across 13 healthy participants for the different shift values in the two motor tasks shown.

In Figure 2 we show how the shift value to be used in the sliding window approach for CMC computation affects the shape of CMC trend along the trial and the timing in CMC-based movement onset detection, considering a representative healthy participant during movements performed with the left hand (similar results were obtained for the right-hand movements). Independently of the shift value, it is worthy of note how the CMC trend accurately tracks the muscular activation as revealed by the EMG signal recorded at the target muscle (ED for Ext movement, FD for Grasp movement), superimposed in each graph (Figures 2A,B, left). CMC resulted as almost null before the EMG onset while it showed an increase and then a plateau around the holding phase of the movement execution. The higher the shift value (updating factor of each sliding window), the more discontinuous the CMC trend appears, as expected since it is obtained for a reduced number of samples. The qualitative comparison between EMG onset and CMC onset in the trends reported in Figure 2 shows how in this representative subject the CMC onset was always delayed with respect to EMG onset and the delay increased with the increase of shift values in both Ext and Grasp conditions. A similar behavior was observed in the other healthy participants. Overall, pie charts (Figures 2A,B, right) reporting the percentages of TD, FD and ND obtained in average across all the healthy participants, show how CMC managed to detect the movement onsets in all movement tasks. Indeed, the averaged percentage of TD across participants (∼88%) considerably overcame the percentages of FD (∼12%) and ND (<1%). FD parameter was the most affected by changes in the shift values, increasing with the increase of the latter (from 9 to 17% in ExtL and from 7 to 15% in GraspL).

The 2-way rmANOVA performed on both TD and MD parameters revealed the SHIFT factor as the only significant effect [TD: *F*(2, 24) = 30.99, *p* < 0.01; MD: *F*(2, 24) = 13.13, *p* < 0.01]. Duncan’s *post-hoc* test applied on TD highlighted a higher value when using the lowest updating factor (125 ms) compared to the others. A significant difference between 250 and 500 ms was also observed.

*Post-hoc* tests applied to MD revealed a significantly higher delay for a shift of 500 ms with respect to other shifts tested. No differences were found between shift of 125 and 250 ms for MD.

**FIGURE 3:**
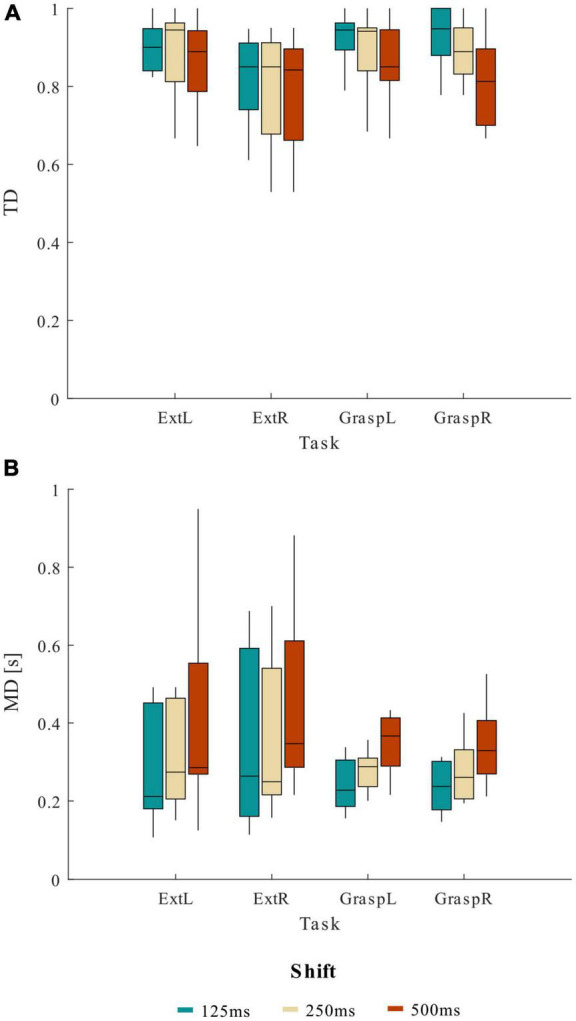
Distribution (boxplots) of **(A)** true detection (TD) and **(B)** mean delay (MD) at the various shift values across 13 healthy participants, separately for the four motor tasks (ExtL, ExtR, GraspL, GraspR).

In Figure 3 we reported the trends of TD (Figure 3A) and MD (Figure 3B) obtained varying the shift values in the sliding window approach for the four movements separately. The lack of significance of the effect MOVEMENT*SHIFT underlines how the shift affected TD and MD parameters independently from the movement type.

Given the results obtained from the statistical analysis, a shift value of 125 ms to update the sliding windows used to compute the CMC was chosen for the pseudo-online movement classification analysis. Using 125 ms of shift resulted on average in TD higher than 82.87% and MD lower than 0.42 s for the four movements tasks.

#### Movement classification in healthy participants

**FIGURE 4:**
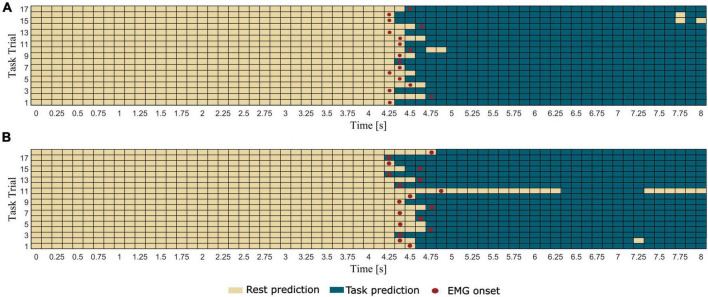
Results of the pseudo-online classification (task vs. rest) performed for all the 57 windows in which each trial was epoched for a representative healthy participant (same as Figure 2) during **(A)** extension and **(B)** grasping of left hand. Rectangles represent the 1 s windows processed by the trained classifier one at a time every 125 ms. Windows predicted by the classifier as rest condition present light color, whereas windows predicted as task condition present dark color. The red dots indicate the first window including the EMG onset (i.e., which ends 125 ms after the EMG onset).

In Figure 4 we report the results of the pseudo-online movement classification performed 1 s window at a time every 125 ms in each testing trial, for a representative healthy participant (same as Figure 2) during extension and grasping of the left hand. It is worth noting how the classifier correctly classified as rest almost all the windows preceding the EMG onset and as movement all the windows succeeding the EMG onset in all the trials. Some misclassifications were found in the movement phase of very few trials where the movement is erroneously classified as rest.

Table 1 summarizes the pseudo-online classification performances across the healthy participants obtained for the different number M of consecutive sliding windows tested as accumulation before a final classification decision was taken. The FNR was null in all four movements, with the exception for GraspR where a FN occurred for one subject in one trial when M was equal to 2 or 3. For all the movements, we obtained on average a hit rate above 88%, with a FPR ranging from 0 to 12% and a delay in the movement detection from 320 to 680 ms according to the value selected for the parameter M. Moreover, the results of the rmANOVA showed how the parameter M significantly affected the classification performances (hit rate, FPR and Mean Delay) in both Ext and Grasp conditions, except for hit rate and FPR in Grasp. As expected, for all the movements, the increasing M led to improved classification accuracy (increase of hit rate and decrease of FPR) but also to an increase of the delay in the detection of movement onset. In particular, the *post-hoc* tests of rmANOVA performed on each classification parameter showed a significant difference between *M* = 1 and *M* = 3 windows and *M* = 1 and *M* = 2 windows. No significant differences were observed in the hit rate and the FPR achieved with *M* = 2 and *M* = 3 windows. Choosing a number M of windows equal to 2 as accumulation before a final movement detection allowed to achieve on average a hit rate higher than 90%, a FPR lower than 10%, and a Mean Delay in the range 470 and 530 ms. Such accumulation factor resulted to be the most promising based on healthy participants’ data.

**TABLE 1:** Pseudo-online classification performances reported as mean ± standard error across 13 healthy participants for each movement task.

Task	Hit rate				FPR				FNR				Mean delay (s)			
	*M* = 1	*M* = 2	*M* = 3	*p*	*M* = 1	*M* = 2	*M* = 3	*p*	*M* = 1	*M* = 2	*M* = 3	*p*	*M* = 1	*M* = 2	*M* = 3	*p*
ExtL	0.89 (±0.04)	0.92 (±0.03)	0.93 (±0.03)	<0.01*	0.11 (±0.04)	0.08 (±0.03)	0.07 (±0.03)	<0.01*	0.00 (±0.00)	0.00 (±0.00)	0.00 (±0.00)	—	0.37 (±0.05)	0.53 (±0.06)	0.68 (±0.06)	<0.01*
ExtR	0.88 (±0.03)	0.90 (±0.03)	0.91 (±0.03)	<0.01*	0.12 (±0.03)	0.10 (±0.03)	0.09 (±0.03)	<0.01*	0.00 (±0.00)	0.00 (±0.00)	0.00 (±0.00)	—	0.34 (±0.04)	0.50 (±0.04)	0.65 (±0.05)	<0.01*
GraspL	0.99 (±0.01)	1.00 (±0.00)	1.00 (±0.00)	—	0.01 (±0.01)	0.00 (±0.00)	0.00 (±0.00)	—	0.00 (±0.00)	0.00 (±0.00)	0.00 (±0.00)	—	0.32 (±0.03)	0.47 (±0.03)	0.61 (±0.03)	<0.01*
GraspR	0.96 (±0.02)	0.97 (±0.02)	0.97 (±0.02)	0.75	0.04 (±0.02)	0.03 (±0.02)	0.03 (±0.02)	0.14	0.00 (±0.00)	0.004 (±0.004)	0.004 (±0.004)	—	0.36 (±0.03)	0.50 (±0.03)	0.67 (±0.04)	<0.01*

Performances are shown for the different number M of consecutive sliding windows tested as accumulation before the final movement detection. The fourth column of each parameter reports the *p*-value of the rmANOVA considering M as within factor. Bold values flanked by asterisks (*) indicate significant differences *p* < 0.01, —ANOVA test not applicable.

Comparing the classification performances obtained with *M* = 2 between the left and the right-hand movements by means of a paired *t*-test (α = 0.05), no significant differences were observed for both Ext (*p* = 0.56) and Grasp (*p* = 0.12) movement.

#### Movement classification in stroke participants

In Table 2 we reported the metrics obtained on varying M parameter with the pseudo-online approach in 12 stroke participants for all the movement types. The four 1-way rmANOVAs performed on the accumulation factor M confirmed what obtained in healthy participants. The FPR increased with increasing M and the *post-hoc* test revealed significant differences between *M* = 1 and *M* = 2 as well as between *M* = 1 and *M* = 3 when the movement was attempted with the paretic hand. A significant difference between the FPR with *M* = 2 and 3 resulted for ExtUH, whereas no significant difference was shown in the FPR for GraspUH. The statistical analysis revealed that in stroke participants the M parameter affected the hit rate only for GraspAH with significant differences between *M* = 1 and *M* = 2 and *M* = 1 and *M* = 3 (no difference was found between the hit rate obtained with *M* = 2 and *M* = 3). As with healthy participants, the higher the M parameter, the greater the Mean Delay. False negatives were more frequent in stroke with respect to healthy participants, in particular for higher M. However, FNR did not exceed 4%.

**TABLE 2:** Pseudo-online classification performances reported as mean ± standard error across 11 stroke participants for Ext movements and 12 stroke participants for Grasp movements.

Task	Hit rate				FPR				FNR				Mean delay (s)			
	*M* = 1	*M* = 2	*M* = 3	*p*	*M* = 1	*M* = 2	*M* = 3	*p*	*M* = 1	*M* = 2	*M* = 3	*p*	*M* = 1	*M* = 2	*M* = 3	*p*
ExtUH	0.81 (±0.05)	0.82 (±0.05)	0.83 (±0.05)	0.75	0.19 (±0.05)	0.16 (±0.05)	0.13 (±0.04)	0.015*	0.00 (±0.00)	0.02 (±0.02)	0.04 (±0.02)	—	0.52 (±0.10)	0.66 (±0.10)	0.88 (±0.13)	<0.01*
ExtAH	0.84 (±0.04)	0.86 (±0.04)	0.87 (±0.04)	0.09	0.15 (±0.04)	0.13 (±0.03)	0.11 (±0.03)	0.05*	0.01 (±0.01)	0.01 (±0.01)	0.02 (±0.01)	—	0.44 (±0.05)	0.62 (±0.07)	0.77 (±0.07)	<0.01*
GraspUH	0.92 (±0.04)	0.93 (±0.03)	0.94 (±0.03)	0.25	0.06 (±0.03)	0.05 (±0.02)	0.04 (±0.02)	0.23	0.01 (±0.01)	0.01 (±0.01)	0.02 (±0.01)	—	0.39 (±0.04)	0.54 (±0.04)	0.70 (±0.05)	<0.01*
GraspAH	0.68 (±0.07)	0.74 (±0.05)	0.79 (±0.05)	<0.01*	0.32 (±0.07)	0.26 (±0.05)	0.21 (±0.05)	<0.01*	0.00 (±0.00)	0.00 (±0.00)	0.004 (±0.004)	—	0.34 (±0.03)	0.50 (±0.03)	0.68 (±0.05)	<0.01*

Performances are obtained considering different number M of consecutive sliding windows tested as accumulation before a final classification decision. The fourth column of each parameter reports the *p*-value of the rmANOVA considering M as within factor. Bold values flanked by asterisks (*) indicate significant differences *p* < 0.01, —ANOVA test not applicable.

Given the results obtained in both healthy and stroke participants, to avoid false positives while maintaining a good timing, the best accumulation factor resulted to be *M* = 2.

**FIGURE 5:**
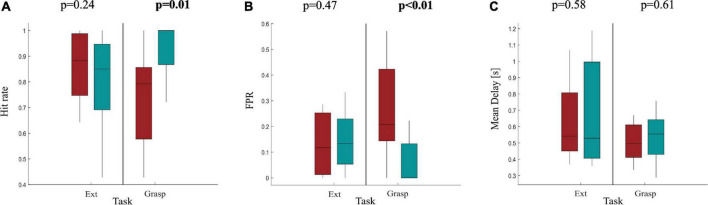
Boxplot diagrams reporting the distributions of **(A)** the Hit rate, **(B)** the FPR and **(C)** the Mean delay in 11 stroke participants as results of the pseudo-online classification using *M* = 2 sliding windows as accumulation before a final classification decision. Performances are reported separately for Ext and Grasp and compared between AH and UH by means of a paired *t*-test (α = 0.05), significant *p*-values are highlighted in bold at the top of each subfigure.

As expected, performances were reduced with respect to those obtained from healthy participants for both movements performed with AH and UH. In Ext condition, for both sides, hit rate was around 84%, the FPR was around 15% while the delay in movement detection was around 580 ms. Differences in terms of classification performances between AH and UH were investigated by means of a statistical analysis whose results are reported in Figure 5. We found a significant difference between AH and UH in Grasp condition only for hit rate and FPR (Figures 5A,B) highlighting how the detection of the grasping movement performed with the affected hand is significantly more difficult with respect to the same movement performed with the unaffected hand but also to the extension movement with both AH and UH. Indeed, in Grasp condition the differences between AH and UH were bigger with respect to Ext movement, with a hit rate significantly lower in Grasp than in Ext for AH (paired *t*-test, *p* = 0.046). Whereas the Mean Delay was approximately the same for AH and UH (Figure 5C).

## Discussion

In this study, we provided evidence that the CMC between brain and muscle activity could discriminate in real-time different hand movements from rest condition in both healthy and stroke participants. The pseudo-online analysis performed on healthy and stroke participants provided information on the parameters representing the best trade-off between classification accuracy and speed when translating CMC computation and its task vs. rest classification from offline to online domain. The testing of such parameters on a stroke participants dataset assessed the feasibility of a CMC-based movement detection in a population of stroke subjects with residual arm activity.

The offline classification performances (Supplementary Tables 2, 3) confirmed the validity of our features extraction and classification approach, previously tested on healthy subjects (Colamarino et al., 2021), also in stroke patients. The high performances obtained from Colamarino and colleagues using the entire set of features (CMC values from all possible EEG-EMG pairs), have been confirmed in this work using as features for movement detection CMC values from only few EEG-EMG electrodes, showing its potential applicability in a clinical setting.

The pseudo-online analysis performed on the shift value showed how the updating factor of CMC computation affects the ability of the CMC to detect the movement. Indeed, it affected both the ability to detect the movement onset (True Detection, TD) and the time to detect it (Mean Delay, MD). Moreover, the pseudo-online classification approach showed how the number of predictions to be accumulated before a given final classification decision affected the pseudo-online classification performance. Classification performance increased according to the number of windows accumulated, and the time to detect the movement with respect to the EMG onset also increased according to it.

One of the main challenges faced by BCI technology is to improve speed and accuracy (Wolpaw et al., 2002; Krauledat et al., 2004; Chai et al., 2014; Thompson et al., 2014; Dagdevir and Tokmakci, 2021) and achieve the reliability necessary for real-word applications (Mcfarland and Wolpaw, 2010). For this reason, identifying the parameters that allow the best trade-off between classification performances and speed is crucial. Over the past decades, many studies have explored feature extraction and classification approaches to improve the accuracy (Bianchi et al., 2022), raise the number of commands (Xu et al., 2020), increase the information transfer rate and reduce the calibration time (Wong et al., 2020; Yao et al., 2022). P300-based speller and steady-state visual evoked potential-based BCIs have mainly taken advantages from those methodological improvements (Nakanishi et al., 2018; Xu et al., 2020) in order to avoid patient frustration caused by false and delayed detections (Eliseyev et al., 2021). However, also in the context of BCIs for rehabilitation, it is crucial to provide an immediate feedback, contingent with the user’s movement intention, in order to re-establish the contingency between cortical activity related to the attempted or imagined movement and the feedback. Indeed, this stimulates the neuroplasticity that leads to motor recovery (Mane et al., 2020; Remsik et al., 2022). In this application, performance improvements were pursued in several ways, e.g., by combining different features such as lateralized readiness potential and event-related desynchronization (Krauledat et al., 2004), refining well-established algorithms of feature extraction and classification and combining them in an innovative way (Lee et al., 2017) or investigating which parameters returned the best performance in terms of both accuracy and timing cost in the ERD/ERS classification (Dagdevir and Tokmakci, 2021).

Although great efforts have been devoted to the optimization of EEG-based BCI, the optimization for the real-time CMC computation and classification has not been investigated yet.

Thanks to the results of our study, we assessed that computing the single-trial CMC every 125 ms and accumulating 2 predictions before a final classification decision allows to achieve good performance (hit rate on average equal to 95 and 84%, FPR on average equal to 5 and 15% for healthy and stroke participants, respectively) and timing (mean delay on average equal to 0.5 s and 0.58 s for healthy and stroke participants, respectively) during two different motor tasks. Meanwhile almost no false negative detections were obtained. The classification accuracy achieved in the present work is higher than that reported in the two available studies using CMC for online control of robotic orthosis in a rehabilitation context (Chowdhury et al., 2019; Guo et al., 2022). Comparable performances were obtained in Chowdhury et al. (2019) when an approach based on statistical correlation is used instead of the classical CMC algorithm. The higher performances obtained in this work could be due to the application of two processing steps helping us to manage the variability in CMC spectral and topographical properties among patients: (i) computation of CMC characteristic frequency in the two EEG-EMG pairs selected for each patient and movement which takes into account inter-patients differences in CMC frequency peak; (ii) application of a feature selection algorithm allowing to select the best EEG-EMG pairs to detect movement, specifically for each patient. No direct comparison can be made between our work and the above-mentioned studies on timing, since they used a different experimental paradigm where the CMC was computed online in a predetermined time interval with respect to the cue and thus the feedback was sent to the patient several seconds after the movement attempt. Hence, this study is the first among those published that analyzed the ability of the CMC in detecting the movement and showed that potentially a CMC-based BCI could send a contingent feedback to the patient right after the attempt.

Moreover, using CMC values as features to discriminate movements from rest condition allows to obtain higher pseudo-online classification performance in stroke patients with respect to previous studies on rehabilitative BCIs based on EEG features only, such as movement related cortical potentials (Mrachacz-Kersting et al., 2016) and sensorimotor rhythms in alpha and beta bands (Lóopez-Larraz et al., 2018), detected during movement attempts.

Beside the main purpose of the presented hBCI (i.e., promoting upper limb motor recovery and avoiding the reinforcement of abnormal muscular activity) its superiority in terms of classification performance can guarantee feedback consistency to patients during training sessions, presumably increasing patients’ satisfaction and motivation toward the ultimate aim, that is a favorable recovery. It is worthy of note that such classification performances are obtained using only fewer features (EEG and EMG channels) compared to those used in previous EEG-based BCI systems during the attempt of motor tasks (Biasiucci et al., 2018; Mrachacz-Kersting and Aliakbaryhosseinabadi, 2018). Thus, our approach appears promising in terms of system usability (computational time, comfort) and set up time, meeting the needs of BCI usage in a clinical context.

Regarding the timing achieved in the classification decision with the parameters selected by this analysis, CMC features were able to provide a fast classification in stroke patients which ensures not only to exploit and train central-to-peripheral communication (Guo et al., 2022), but also to send ecological feedback to the patient right after the onset of the movement attempt (a feedback that is congruent in timing and content with the exercise setting), favoring an effective motor re-learning. Comparing classification speed with previous works on rehabilitative BCIs during movement attempts, we obtained comparable (Mrachacz-Kersting and Aliakbaryhosseinabadi, 2018) or better (Biasiucci et al., 2018, 3.5–5 s to deliver feedback) results with respect to EEG-based approaches.

Furthermore, comparing the performances of the affected and unaffected hand in stroke participants confirmed how the CMC is affected by stroke (Krauth et al., 2019) in particular during the grasping task, the only case in which significant differences were obtained between the two sides. Similar to what reported in our previous work on healthy subjects (Colamarino et al., 2021), the extension task was easier to detect by means of CMC features also in stroke patients.

Despite the promising results obtained on both healthy and stroke participants by applying the CMC-based approach in movements detection, the performances obtained by means of a pseudo-online approach should be confirmed by online experiments. In fact, the exclusion of both non-compliant and artefactual trials from the analysis before CMC computation might have mildly overestimated the classification performances since they were calculated on data with a higher signal-to-noise ratio. However, we are confident that such overestimation effect is limited since we rejected around one/two trials out of the 20 requested per condition. In the online implementation of our approach, non-compliance will be manually checked by the therapist/experimenter who will start a new trial (request of movement attempt) only when the muscle activation level will be below the desired threshold or terminate the trial before the established duration if the patient is not performing the task (i.e., non-compliant trials should virtually never occur).

To the best of our knowledge, this is the first study that tested the ability of CMC features to detect in real-time movement attempts in stroke patients with particular focus on the best parameters to use in the computation to ensure an accurate and fast detection. The results obtained here stated the feasibility of CMC features as inputs of a h-BCI for upper limb motor rehabilitation. Such a CMC-based BCI would allow to exploit the patients’ residual or recovered arm activity, filling the gap between the early stage of rehabilitation when severely disabled patients (i.e., plegic) can only imagine the movements during a BCI-based training intervention (Pichiorri et al., 2015) and the progressive functional recovery. This would allow to follow patients along each stage of their rehabilitation path with a strategy tailored to their level of impairment and hence maximizing the time and amount of functional recovery.

Our results should be confirmed enlarging the number of stroke participants involved in the study and including patients with different degrees of impairment. Moreover, assessment of BCI performances in stroke patients during online BCI training sessions is needed to confirm the feasibility of our approach, including an evaluation of usability, satisfaction and workload of patients and professionals in a real-world setting. Further studies should focus on which CMC features should be reinforced during the h-BCI training, and which ones should be discouraged to avoid the maladaptive movement abnormalities typical of post-stroke recovery such as spasticity, co-contractions and motor overflow (Levin et al., 2000; Miller and Dewald, 2012; Silva et al., 2014; Chen et al., 2018). Lastly, the clinical efficacy of such a BCI must be validated in a Randomized Control Trial in stroke patients, with particular focus on motor function recovery and muscular control abnormalities.

## Conclusion

This study introduces an optimized approach to employ CMC features for the real-time detection of different hand movements in a h-BCI. Such a CMC-based h-BCI would be able to re-establish impaired CMC, achieving a good timing and accuracy crucial for patients’ motor re-learning and motivation during the rehabilitation training. The results achieved in this analysis will ground the design of a novel non-invasive h-BCI in which the control feature is derived from a combined EEG and EMG connectivity pattern estimated during upper limb movement attempts. Such device would have a potential high impact on the future design of novel stroke rehabilitation strategies.

## Data availability statement

The raw data supporting the conclusions of this article will be made available by the authors, without undue reservation.

## Ethics statement

The studies involving human participants were reviewed and approved by the Ethical Committee of the IRCCS Santa Lucia Foundation, Rome, Italy (CE PROG.752/2019). The patients/participants provided their written informed consent to participate in this study.

## Author contributions

VS: implementation of experimental procedure, EEG data collection, implementation and validation of data analysis, and manuscript writing. JT: implementation and validation of experimental procedure, EEG-EMG data analysis supervision, results interpretation, and manuscript writing supervision. EC: implementation of experimental procedure, EMG data collection, and analysis supervision. RM: EEG-EMG data analysis support. FCa: pseudo-online classification analysis support. FCi: validation of experimental procedure and overall data analysis supervision. DM: validation of experimental procedure, overall data interpretation, and manuscript writing supervision. FP: responsible for study, design of experimental procedure, results interpretation, and manuscript writing supervision. All authors contributed to the article and approved the submitted version.

## Footnotes

1
https://www.brainproducts.com

2
https://www.cometasystems.com

3
http://psychtoolbox.org
