# Supplementary material for: Cortico-muscular coupling to control a hybrid brain-computer interface for upper limb motor rehabilitation: A pseudo-online study on stroke patients
Source: Front Hum Neurosci. 2022 Nov 22;16:1016862. doi: 10.3389/fnhum.2022.1016862 (PMC9722732; doi:10.3389/fnhum.2022.1016862)
Supplement: Supplementary file 1 [file Data_Sheet_1.docx]

**Cortico-Muscular Coupling to control a hybrid Brain-Computer Interface for upper limb motor rehabilitation: a pseudo-online study on stroke patients**

**Valeria de Seta^1,2^, Jlenia Toppi^1,2*^, Emma Colamarino^1,2^, Rita Molle^2^, Filippo Castellani^2^, Febo Cincotti^1,2^, Donatella Mattia^2^, Floriana Pichiorri^2^**

^1^ Department of Computer, Control, and Management Engineering, Sapienza University of Rome, Rome, Italy, ^2^ Neuroelectric Imaging and BCI Lab, IRCCS Fondazione Santa Lucia, Rome, Italy

Supplementary Material

# Supplementary Tables

**Supplementary Table 1** - Demographic and clinical information of stroke participants. FMA-UE=Fugl-Meyer Assessment scale upper extremity section ranging from 0 (most affected) to 66 (least affected); FMA A = upper-limb subscale; FMA B = wrist subscale; FMA C = hand subscale; FMA D = coordination/speed subscale; MAS=Modified Ashworth Scale; NIHSS=National Institute of Health Stroke Scale.

| **ID** | **Age** | **Gender** | **Handedness** | **Months from event** | **Type** | **Lesion type** | **Lesion side** | **FMA-UE** | **FMA A** | **FMA B** | **FMA C** | **FMA D** | **NIHSS** | **MAS** |
| --- | --- | --- | --- | --- | --- | --- | --- | --- | --- | --- | --- | --- | --- | --- |
| P1 | 76 | M | Right | 3 | subacute | ischemic | left | 51 | 31 | 3 | 11 | 6 | 1 | 0 |
| P2 | 73 | M | Right | 13 | chronic | ischemic | left | 63 | 35 | 8 | 14 | 6 | 3 | 0 |
| P3 | 59 | M | Right | 5 | subacute | hemorrhagic | left | 23 | 14 | 2 | 7 | 0 | 4 | 2 |
| P4 | 75 | F | Right | 3 | subacute | ischemic | right | 62 | 33 | 10 | 14 | 5 | 4 | 0 |
| P5 | 53 | F | Right | 4 | subacute | hemorrhagic | left | 57 | 32 | 9 | 11 | 5 | 3 | 0 |
| P6 | 21 | F | Right | 3 | subacute | hemorrhagic | right | 54 | 29 | 8 | 13 | 4 | 2 | 1 |
| P7 | 55 | M | Left | 5 | subacute | hemorrhagic | right | 52 | 30 | 7 | 11 | 4 | 0 | 2 |
| P8 | 40 | F | Right | 11 | chronic | hemorrhagic | right | 58 | 30 | 10 | 13 | 5 | 0 | 0 |
| P9 | 24 | F | Right | 8 | chronic | ischemic | left | 57 | 31 | 7 | 14 | 5 | 2 | 0 |
| P10 | 51 | M | Right | 4 | subacute | hemorrhagic | left | 26 | 16 | 3 | 7 | 0 | 3 | 4 |
| P11 | 60 | M | Right | 2 | subacute | ischemic | left | 42 | 26 | 6 | 7 | 3 | 2 | 1 |
| P12 | 59 | M | Right | 3 | subacute | ischemic | right | 48 | 28 | 3 | 13 | 4 | 4 | 4 |

**Supplementary Table 2** - Offline task-vs-rest classification performances reported as mean ± standard error across 13 healthy participants. ExtR: finger extension with the right hand; ExtL: finger extension with the left hand; GraspR: grasping with the right hand; GraspL: grasping with the left hand.

| **Task** | **AUC** | **Accuracy** | **Sensitivity** | **Specificity** |
| --- | --- | --- | --- | --- |
| ExtL | 0.98±0.01 | 0.93±0.02 | 0.90±0.02 | 0.97±0.01 |
| ExtR | 0.99±0.004 | 0.94±0.02 | 0.91±0.02 | 0.97±0.01 |
| GraspL | 1.00±0.002 | 0.97±0.01 | 0.94±0.01 | 0.99±0.01 |
| GraspR | 0.99±0.004 | 0.95±0.01 | 0.91±0.02 | 0.99±0.01 |

**Supplementary Table 3** - Offline task-vs-rest classification performances reported as mean ± standard error across 11 stroke participants for Ext movements and 12 stroke participants for Grasp movements. ExtUH: finger extension with the unaffected hand; ExtAH: finger extension with the affected hand; GraspUH: grasping with the unaffected hand; GraspAH: grasping with the affected hand.

| **Task** | **AUC** | **Accuracy** | **Sensitivity** | **Specificity** |
| --- | --- | --- | --- | --- |
| ExtUH | 0.93±0.03 | 0.88±0.03 | 0.83±0.05 | 0.93±0.02 |
| ExtAH | 0.98±0.01 | 0.92±0.03 | 0.88±0.04 | 0.96±0.02 |
| GraspUH | 0.95±0.03 | 0.91±0.03 | 0.84±0.06 | 0.99±0.01 |
| GraspAH | 0.95±0.03 | 0.90±0.03 | 0.85±0.05 | 0.96±0.02 |
